# Supplementary material for: Incidence and Risk Factors of Venous Thromboembolic Events in Patients with ANCA-Glomerulonephritis: A Cohort Study from the Maine-Anjou Registry
Source: J Clin Med. 2020 Sep 30;9(10):3177. doi: 10.3390/jcm9103177 (PMC7599765; doi:10.3390/jcm9103177)
Supplement: Supplementary file 1 [file jcm-09-03177-s001.pdf]

Table S1. Search for a hypercoagulability state. Results are given as absolute values and percentage.

	***n* (%)**
Search for anticardiolipid antibodies	64 (48.1)
Positivity of IgG *	2 (3.1)
Positivity of IgM *	3 (4.7)
Search for anti-beta2 GP1 antibodies	55 (41.3)
Positivity of IgG *	0 (0.0)
Positivity of IgM *	0 (0.0)
Search for LA	41 (30.8)
Positivity of LA *	6 (14.6)
At least one positive test **	8 (11.9)

LA, Lupus anticoagulants; * Among screened patients; ** Among patients tested for at least one test (*n* = 67).

Table S2. Baseline characteristics of patients according to statin treatment at diagnosis.

	**Statin Therapy**		
	**Yes, *n* = 49**	**No, *n* = 84**	***p***
**Baseline characteristics**			
Gender (M/F)	34/15	50/34	0.255
Age (years)	64.6 ± 11.5	65.3 ± 15.5	0.747
BMI (Kg/m^2)	25.0 ± 5.7	24.3 ± 3.7	0.500
Hypertension, *n* (%)	28 (57.1)	44 (52.4)	0.595
Diabetes mellitus, *n* (%)	8 (16.3)	10 (11.9)	0.472
History of VTE, *n* (%)	1 (2.0)	7 (8.3)	0.141
**ANCA-associated vasculitis characteristics**			
**Clinical diagnosis, *n* (%)**			
GPA/MPA, *n* (%)	21 (42.9)/28 (57.1)	60 (71.4)/24 (28.6)	0.093
Newly diagnosed, *n* (%)	6 (12.2)	5 (5.9)	0.204
**ANCA type, *n* (%)**			0.767
PR3-ANCA	15 (30.6)	24 (28.6)	-
MPO-ANCA	32 (65.3)	54 (64.3)	-
ANCA negative	2 (4.1)	6 (7.1)	-
**BVAS at AAV diagnosis or relapse**	17.1 ± 6.3	17.3 ± 6.0	0.869
**Organ involvement at diagnosis, *n* (%)**			
Cutaneous signs	10 (20.4)	15 (17.9)	0.741
Ear, nose, throat	21 (42.9)	25 (29.8)	0.126
Heart	2 (4.1)	6 (7.1)	0.710
Digestive	2 (4.1)	5 (6.0)	1.000
Lung	19 (38.8)	29 (34.5)	0.662
Renal (at AAV diagnosis or relapse)			
Serum creatinine, μmol/L, median (min-max)	290.0 (77–1007)	240.0 (30–1445)	0.425
eGFR, mL/min/1.73 m^2, median (min-max)	19.8 (5–92)	22.7 (5–200)	0.759
Proteinuria/creatinunuria, g/g, median (min-max)	2.2 (0.3–5.3)	1.24 (0.7–14.0)	0.137
Need for renal replacement therapy, *n* (%)	11 (22.4)	19 (22.6)	0.982
Neurological	6 (12.2)	13 (14.6)	0.589
**Biology at AAV diagnosis or relapse**			
C-reactive protein, mg/L, median (min-max)	76 (3–346)	46.5 (1–319)	0.402
Serum albumin, g/L	29.2 ± 5.7	29.1 ± 6.7	0.970
**Treatment at AAV diagnosis or relapse, *n* (%)**			
Antiplatelet agents	22 (44.9)	9 (10.7)	<0.001
Anticoagulant therapy	6 (12.2)	11 (13.1)	0.887
**Lipid concentration, g/L ***			
Total cholesterol	2.09 ± 0.6	2.13 ± 0.7	0.781
LDL cholesterol	1.20 ± 0.4	1.20 ± 0.5	0.990
HDL cholesterol	0.58 ± 0.3	0.58 ± 0.2	0.945

* Available in 94 patients at AAV diagnosis.

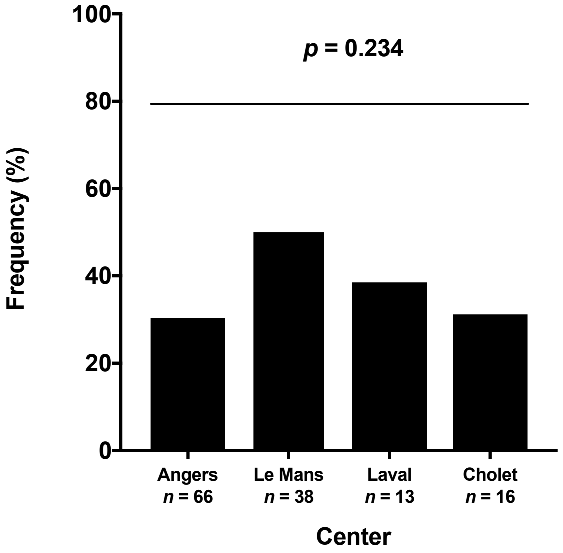

Figure S1. Statin treatment frequency according to center.
